# Ecto-5′-Nucleotidase (CD73) Regulates the Survival of CD8+ T Cells

**DOI:** 10.3389/fcell.2021.647058

**Published:** 2021-04-13

**Authors:** Mariana V. Rosemblatt, Brian Parra-Tello, Pedro Briceño, Elizabeth Rivas-Yáñez, Suat Tucer, Juan Saavedra-Almarza, Pilar Hörmann, Brandon A. Martínez, Álvaro Lladser, Mario Rosemblatt, Caglar Cekic, María Rosa Bono, Daniela Sauma

**Affiliations:** ^1^Departamento de Biología, Facultad de Ciencias, Universidad de Chile, Santiago, Chile; ^2^Facultad de Medicina y Ciencia, Universidad San Sebastián, Santiago, Chile; ^3^Department of Molecular Biology and Genetics, Bilkent University, Ankara, Turkey; ^4^Fundación Ciencia & Vida, Santiago, Chile

**Keywords:** CD73/NT5E, homeostatic, CD8+ T cell, antigenic activation, CD127 (IL7 receptor), CD25

## Abstract

Ecto-5′-nucleotidase (CD73) is an enzyme present on the surface of tumor cells whose primary described function is the production of extracellular adenosine. Due to the immunosuppressive properties of adenosine, CD73 is being investigated as a target for new antitumor therapies. We and others have described that CD73 is present at the surface of different CD8+ T cell subsets. Nonetheless, there is limited information as to whether CD73 affects CD8+ T cell proliferation and survival. In this study, we assessed the impact of CD73 deficiency on CD8+ T cells by analyzing their proliferation and survival in antigenic and homeostatic conditions. Results obtained from adoptive transfer experiments demonstrate a paradoxical role of CD73. On one side, it favors the expression of interleukin-7 receptor α chain on CD8+ T cells and their homeostatic survival; on the other side, it reduces the survival of activated CD8+ T cells under antigenic stimulation. Also, upon *in vitro* antigenic stimulation, CD73 decreases the expression of interleukin-2 receptor α chain and the anti-apoptotic molecule Bcl-2, findings that may explain the reduced CD8+ T cell survival observed in this condition. These results indicate that CD73 has a dual effect on CD8+ T cells depending on whether they are subject to an antigenic or homeostatic stimulus, and thus, special attention should be given to these aspects when considering CD73 blockade in the design of novel antitumor therapies.

## Introduction

Current immunotherapies against cancer consist of the adoptive transfer of *ex vivo* tumor-specific CD8+ T cells to the patient; however, these therapies are not always successful (Restifo et al., 2012). Several strategies have emerged to increase the efficacy of adoptive cell transfer therapies, such as prior lymphocyte depletion (lymphoconditioning) to eliminate immunosuppressive cells, immune checkpoint inhibition, or modulation of the differentiation status of transferred CD8+ T cells (Gattinoni et al., 2006; Restifo et al., 2012). It has been reported that the degree of differentiation of CD8+ T cells used during antitumor therapy is inversely correlated with their ability to persist in the tumor microenvironment and, consequently, eradicate tumors (Gattinoni et al., 2006). In this line, the adoptive transfer of highly differentiated lymphocytes, such as effector CD8+ T cells, has shown to be disadvantageous in the long term (Gattinoni et al., 2005). These observations raise the concern about using terminally differentiated CD8+ T cells for adoptive cell therapy and highlight the relevance of understanding the mechanisms that drive CD8+ T cell differentiation into different subsets.

Activation of naïve CD8+ T cells enables the growth and generation of heterogeneous populations, which differ in proliferative potential, maintenance, survival, and cytotoxic capacity (Gattinoni et al., 2012). Among these populations, effector T lymphocytes present a higher differentiation status, which translates into higher levels of expression of the alpha chain of IL-2 receptor (CD25) in addition to having a high proliferative and cytotoxic capacity (Kalia et al., 2010; Zhang and Bevan, 2011; Boyman and Sprent, 2012). On the other hand, naïve and memory cells produce low levels of effector proteins but present a higher survival and self-renewal capacity than effector cells, and thus have been considered for use in immunotherapies against cancer (Fearon et al., 2001; Gattinoni et al., 2011).

In the absence of antigenic stimulation, homeostatic cytokines such as IL-15 and IL-7 allow the maintenance of memory and naïve T cells (Tan et al., 2001, 2002; Goldrath et al., 2002; Raeber et al., 2018). IL-7 is produced by a subset of fibroblastic reticular cells within secondary lymphoid organs and is retained in these sites by binding to the extracellular matrix (Link et al., 2007; Barata et al., 2019). T cell response to IL-7 is regulated through the modulation of the IL-7 receptor alpha chain (IL-7Rα or CD127), which dimerizes with the common gamma chain to form the IL-7 receptor (Kondo et al., 1994). CD127 is mainly expressed by naïve and memory T cells (Schluns et al., 2000), and its expression is regulated by IL-7 and T cell receptor (TCR) signaling (Raeber et al., 2018). IL-7/CD127 signaling axis enables the survival and maintenance of T cells by promoting the expression of the anti-apoptotic Bcl-2 protein, preventing apoptosis via the mitochondrial pathway (Akashi et al., 1997; Schluns et al., 2000; Wojciechowski et al., 2007; Surh and Sprent, 2008; Jacobs et al., 2010). Although memory T cells also rely on IL-7 for survival, IL-15 is the main cytokine driving their homeostatic proliferation (Surh and Sprent, 2000; Raeber et al., 2018).

In contrast to naïve T cells, antigen-activated T cells are short-lived, and the factors that promote their *in vivo* survival are not fully elucidated. CD127 is downregulated upon antigenic stimulation, and T cell numbers are controlled by other mechanisms that include CD25/IL-2 signaling (Marrack et al., 2000) and the activation of the PI3-kinase/Akt pathway (Xue et al., 2002). Moreover, prolonged IL-2 signaling promotes terminal effector differentiation of CD8+ T cells (Pipkin et al., 2010).

Adenosine is considered an immunosuppressive molecule, which reduces the activation and proliferation of effector lymphocytes through the A2A receptor (A2AR) signaling (Linnemann et al., 2009; Mastelic-Gavillet et al., 2019). We have demonstrated that the adenosine/A2AR axis is relevant for the maintenance and survival of naïve lymphocytes under homeostatic proliferation conditions since it prevents the down-regulation of CD127 in CD8+ T cells. Interestingly, adenosine signaling showed no effect on the maintenance of central memory lymphocytes (Cekic et al., 2013), suggesting an additional regulatory mechanism for the survival of memory cells.

The main enzyme involved in adenosine production is ecto-5′-nucleotidase (CD73), and in consequence, it is currently considered a novel immune checkpoint (Allard et al., 2016; Chen et al., 2019). CD73 is expressed by tumor cells and also by immune populations such as naïve and memory CD8+ T cells (Bono et al., 2015). However, the role of this enzyme in the differentiation, maintenance, and survival of CD8+ T cells has not been addressed. Here we describe that CD73 has a dual effect on CD8+ T cells depending on whether T cells are subject to homeostatic or antigenic stimulation. Using murine models of homeostatic and antigenic conditions, we demonstrate that CD73 favors the expression of interleukin-7 receptor α chain on CD8+ T cells and their homeostatic survival. In contrast, CD73 reduces the survival of activated CD8+ T cells under antigenic stimulation, a finding that may be explained through the downregulation of the expression of the interleukin-2 receptor α chain and the anti-apoptotic molecule Bcl-2. These results indicate that the role of CD73 on CD8+ T cells should be considered when designing effective antitumor therapies.

## Materials and Methods

### Mice

CD73KO (B6.129S1-Nt5e^tm1Lft^/J), C57BL/6 (CD45.2+), CD45.1+ (B6.SJL-Ptprc^a^ Pepc^b^/BoyJ), Rag1–/– (B6.129S7-Rag1^tm1Mom^/J), and OT-I (C57BL/6-Tg (TcraTcrb)1100Mjb/J) mice were purchased from Jackson Laboratory. OT-I/CD73KO mice were obtained by backcrossing the F1(OT-IxCD73KO) with CD73KO mice and testing for Vα2Vβ5 transgenic TCR and CD73 expression by FACS. The mice were kept in the animal facility at Fundacion Ciencia & Vida. Animal work was carried out under institutional regulations of Fundacion Ciencia and Vida and Facultad de Ciencias, Universidad de Chile and was approved by the local ethics review committees. Usually, 6–8 weeks old mice were used for all experiments except for those used to obtain central memory CD8+ T cells.

### Antibodies

Antibodies against CD8a PECy7 (clone 53-6.7), CD8a APCeFluor780 (clone 53-6.7), CD25 PE (clone PC61.5), CD62L FITC (clone MEL-14), Vα2 PE (clone B20.1) and Vβ5 FITC (clone MR9-4) were obtained from eBioscience. Antibodies against CD44 APC (clone IM7), CD45.1 PECy7 (clone A20), CD45.2 Alexa Fluor 647 (clone 104), CD127 APC (clone A7R34), Ki-67 Alexa fluor 488 (clone 11F6), Bcl-2 PE (clone BCL/10C4), CD16/32 (FcBlock) and activating antibodies α-CD3 (clone 145- 2C11) and α-CD28 (clone 37.51) were obtained from BioLegend.

### Flow Cytometry

For cell surface staining, cellular suspensions were incubated in the dark for 30 min at 4°C with a mixture of antibodies conjugated with different fluorochromes in the presence of a viability dye (Fixable Viability Dye eFluor 780, eBiosciencies) to discard dead cells. The cells were then centrifuged at 600 × *g* for 7 min at 4°C and resuspended in PBS + 2% FBS for FACS analysis.

To assess the frequency and absolute numbers of transferred CD8+ T cells, blood was drawn at different time points by cutting the lateral tail vein of the mice with a scalpel. Blood was collected in heparinized tubes (Sanderson Laboratory, 1,000 I.U./mL) and then incubated with antibodies against CD16/CD32 (Fc Block) before the addition of a mixture of fluorochrome-conjugated antibodies. Blood was incubated at room temperature for 20 min and then lysed with 1 ml of red blood cell lysis solution (BD FACS lysing solution, BD Biosciences). The sample was washed and the pellet resuspended in PBS + 2% FBS for FACS analysis. Absolute numbers of CD8+ T cells in blood were analyzed using the CountBright Absolute Counting Beads (Life Technologies) following the manufacturer's instructions.

To analyze the intracellular expression of Bcl-2 and Ki-67, following cell-surface staining, cells were fixed and permeabilized with Foxp3 Fixation/Permeabilization Buffer (eBiocience) and incubated for 20 min at 4°C. Next, the cells were washed with Permeabilization Buffer (BD Biosciences) and incubated with anti-Bcl-2 or Ki-67 antibodies for 30 min at 4°C. Finally, the cells were washed with Permeabilization Buffer and resuspended in PBS + 2% FBS for FACS analysis. FACS data were analyzed using the FlowJo program V10 (Tree Star, Inc.).

### Annexin V/PI Staining

Cells were harvested, centrifuged at 600 × *g* for 7 min and resuspended in 100 μl of Binding Buffer (10 mM HEPES, 140 mM NaCl, 2.5 mM CaCl2, and pH 7.4) containing 0.5 μl of Annexin V APC (Biolegend) and 2 μl of propidium iodide (50 μg/ml) (Sigma). Cells were incubated for 20 min at room temperature and finally, 300 μl of Binding Buffer was added. Cells were immediately analyzed by FACS.

### Cell Trace Violet (CTV) Staining

For CTV labeling, cells are washed and resuspended in phosphate-buffered saline (PBS) at 10^7^ cells/ml in the presence of CTV (10 μM). They were incubated for 20 min at 37°C in the dark. The reaction was stopped by adding 5 mL of IMDM + 10% FBS and incubating for 5 min. The cells were subsequently washed and counted in a Neubauer chamber, excluding dead cells using trypan blue dye.

### Isolation of Naïve and Central Memory CD8+ T Cells

Naïve and central memory CD8+ T cells were obtained from spleen and peripheral lymph nodes (PLN) of WT (CD45.1+), CD73KO (CD45.2+), OT-I (CD45.1+/CD45.2+), or OT-I/CD73KO (CD45.2+) mice. Briefly, the spleens were perfused with RPMI 1640 supplemented with 10% FBS. Lymph nodes were mechanically disaggregated with scissors. The cell suspension was filtered through a metal mesh, and CD8+ T cells were enriched by negative selection using MACS magnetic beads (Miltenyi Biotec) following the manufacturer's instructions. After CD8+ T cell enrichment, naïve CD8+ T cells (CD8+/CD44low/CD62Lhigh/CD25–) and central memory CD8+ T cells (CD8+/CD44+/CD62Lhigh/CD25–) were obtained by cell sorting using a FACS Aria III cell sorter (Biosciences).

### *In vivo* Homeostatic Proliferation

Naïve cells from WT and CD73KO mice were counted under a microscope with trypan blue staining and mixed at a 1:1 ratio. The initial ratio or input was analyzed by FACS using antibodies against CD45.1 and CD45.2. A total of 10^6^ total cells from this mixture were intravenously (i.v.) transferred into Rag1–/– mice. On days 7, 14, 21, and 28 after the adoptive transfer, blood samples were drawn and analyzed by FACS. On day 28, the mice were euthanized, and the transferred cells were analyzed in the spleen, PLN, and MLN by FACS. The following formula was used to obtain the CD73KO/WT ratio:

$$\begin{array}{l} \text{CD}73\text{KO}/\text{WT ratio}\\ =\left(\text{CD}73\text{K}{\text{O}}_{\text{organ}}/\text{W}{\text{T}}_{\text{organ}}\right)/\left(\text{CD}73\text{K}{\text{O}}_{\text{input}}/\text{W}{\text{T}}_{\text{input}}\right). \end{array}$$

### *In vivo* Antigenic Proliferation

Naïve CD8+ T lymphocytes obtained from OT-I (CD45.1+/CD45.2+) and OT-I/CD73KO (CD45.2+) mice were counted in a Neubauer chamber under the microscope with trypan blue staining and mixed at a 1:1 ratio. The initial ratio (input) was then analyzed by FACS using antibodies against CD45.1 and CD45.2. A total of 1 × 10^6^ cells from this mixture were adoptively transferred (i.v.) into CD45.1+ mice. One day after the adoptive transfer, the mice were intraperitoneally (i.p.) immunized with 500 μg of OVA protein (Sigma-Aldrich) and 50 μg of Poly I:C (InvivoGen). On days 7, 14, 21, and 28 after adoptive transfer blood samples were obtained and analyzed by FACS. On day 28, mice were euthanized, and the transferred cells were analyzed by FACS in the spleen, PLN, and mesenteric lymph node (MLN). The following formula was used to obtain the CD73KO/WT ratio:

$$\begin{array}{l} \text{CD}73\text{KO}/\text{WT ratio}\\ =\left(\text{CD}73\text{K}{\text{O}}_{\text{organ}}/\text{W}{\text{T}}_{\text{organ}}\right)/\left(\text{CD}73\text{K}{\text{O}}_{\text{input}}/\text{W}{\text{T}}_{\text{input}}\right). \end{array}$$

For *in vivo* proliferation assays, naïve CD8+ T cells from WT and CD73KO mice were labeled with CTV (10 μM) and counted in a Neubauer chamber under the microscope excluding dead cells by trypan blue staining. Cells were then mixed at a 1:1 ratio. The initial ratio (input) was then evaluated by FACS using antibodies against CD45.1 and CD45.2. A total of 1 × 10^6^ cells from this mixture were adoptively transferred (i.v.) into CD45.1+ mice. One day after the adoptive transfer, the mice were i.p. immunized with 500 μg of OVA protein (Sigma-Aldrich) and 25 μg of lipopolysaccharide (LPS, Sigma). On day 4 the mice were euthanized, and the transferred cells were analyzed by FACS in the blood, the spleen, PLN, and MLN.

### *In vitro* Culture

Naïve CD8+ T cells were obtained from the spleen and PLN from WT or CD73KO mice as previously described. The cells were labeled with CTV, cultured in a 96-well round-bottom microplate (10^5^ CD8^+^ T cells/ well) and activated with soluble α-CD3 (1 μg/ml; clone 145- 2C11, eBioscience) and α-CD28 (1 μg/ml; clone 37.51) for 4 days in the presence of 10 ng/ml recombinant mouse IL-2 (eBioscience). In some experiments, the CD73 inhibitor APCP [Adenosine 5'-(a,b-methylene) diphosphate] was added at 50 μM (Sigma-Aldrich) to the cultures. When indicated, the stable, cell-impermeable analog of adenosine NECA (Tocris) was added to the cultures at 1 μM in the presence of 1 U/mL adenosine deaminase (Roche) to prevent the effect of endogenously generated adenosine. The selective A2AR antagonist SCH58261 (Sigma-Aldrich) was added at a final concentration of 5 μM.

### Statistical Analysis

Data are expressed as mean ± standard deviation (SD). The comparison of the mean fluorescence intensity and cell frequency between two groups was performed with the non-parametric Mann-Whitney test or the parametric t-Test if the samples followed a normal distribution. For the comparison of three or more data groups, the Kruskal-Wallis test or one-way ANOVA was used if the samples followed a normal distribution. For the statistical analysis of absolute numbers of T cells in homeostatic proliferation experiments, a two-way ANOVA test and Bonferroni post-test were used. The GraphPad Prism 5 program (GraphPad Software, San Diego, CA, USA) was used for the statistical analysis. Statistical significance was considered with a value of *p* < 0.05. (^*^p < 0.05, ^**^p < 0.01, ^***^ *p* < 0.005, ^****^ *p* < 0.001).

## Results

### CD73 Promotes Homeostatic Proliferation and CD127 Expression on Naïve CD8+ T Cells

**Figure 1:**
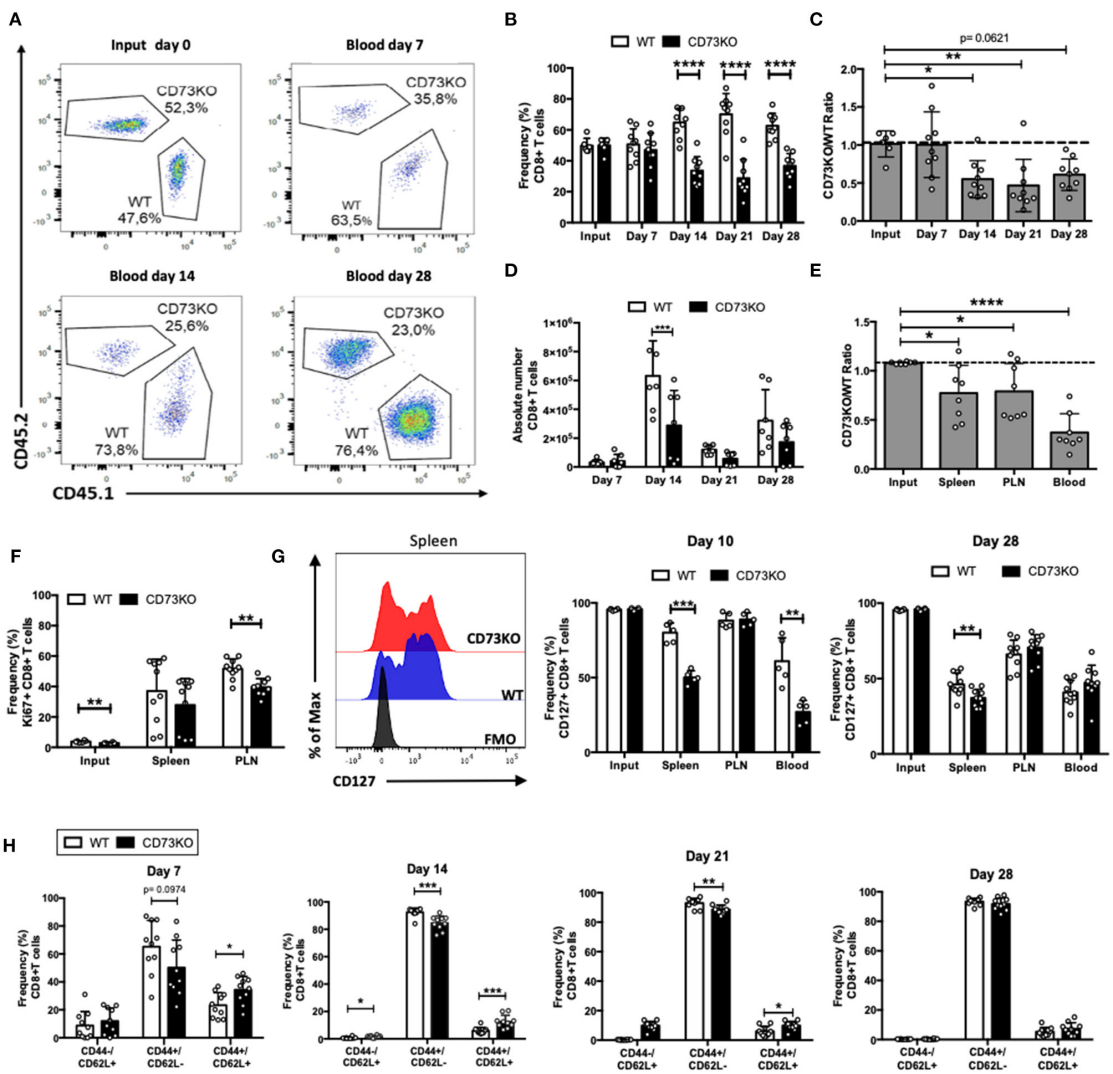
CD73 promotes *in vivo* homeostatic proliferation and CD127 expression in naïve CD8+ T cells. Naïve CD8+ T cells (CD8+/CD25-/CD44-/CD62L+) obtained from WT (CD45.1+) and CD73KO (CD45.2+) mice were co-transferred in Rag1–/– mice. Rag1–/– mice were bled at different days to analyze the frequency and phenotype of transferred cells. Mice were euthanized on day 28 to analyze the transferred cells in the spleen and peripheral lymph nodes (PLN). **(A)** FACS plots showing the input and frequency of transferred cells at days 7, 14, and 28 in the blood**. (B)** Frequency of transferred cells in the blood at input and days 7, 14, 21, and 28. **(C)** CD73KO/WT ratio at input and days 7, 14, 21, and 28 in blood. **(D)** Absolute numbers of transferred CD8+ T cells in the blood at days 7, 14, 21, and 28. **(E)** CD73KO/WT ratio at input and day 28 in the spleen, PLN, and the blood. **(F)** Frequency of Ki67+ cells (gated on transferred cells) at input, spleen and PLN at day 28. **(G)** Left, FACS histogram overlay depicting CD127 expression (gated on transferred cells) in the spleen at day 28. Right, frequency of CD127+ among transferred cells in the spleen, PLN and the blood at day 10 and 28. **(H)** Frequency of transferred cells expressing naïve (CD44-/CD62L+), effector memory (CD44+/CD62L–), and central memory (CD44+/CD62L+) phenotypes at days 7, 14, 21, and 28 in the blood. All data represent mean ± s.d. Data were analyzed by two-tailed unpaired Student's *t*-Test **(B,D, F–H)** or one-way ANOVA with Bonferroni post-hoc test **(C,E)**. *p < 0.05; **p < 0.01; ***p < 0.005; ****p < 0.001.

We have previously demonstrated that adenosine promotes the survival of naïve T cells (Cekic et al., 2013). As CD73 is the main enzyme involved in adenosine production, we first sought to analyze the role of this ectonucleotidase in naïve CD8+ T cell homeostatic survival. For this, we co-transferred sorted naïve WT (CD45.1+) and CD73KO (CD45.2+) CD8+ T cells (CD8+/CD25-/CD44-/CD62Lhi) into Rag1–/– recipient mice, and we analyzed the frequency and the absolute number of these cells in the blood at different time points. As shown in Figures 1A,B, the frequency of CD73KO CD8+ T cells in the blood at day 14 was reduced compared with WT CD8+ T cells, and this difference was maintained until day 28 following the transfer. Accordingly, the ratio of CD73KO/WT cells at days 14 and 21 following the transfer was reduced compared to the input (Figure 1C). In this homeostatic condition, WT CD8+ T cells suffered approximately a six-fold increase in the absolute numbers at day 14, while CD73KO CD8+ T cells suffered only approximately a three-fold increase in their absolute number (Figure 1D). This difference is not due to a preferential migration or retention of CD73KO CD8+ T cells in other organs, as we observed a reduced CD73KO/WT cell ratio in the spleen and peripheral lymph nodes (PLN) as well (Figure 1E).

Next, we analyzed whether this decrease in the number of CD73KO cells was due to a reduced proliferative capacity. For this, we analyzed Ki67 expression in the transferred CD8+ T cells at day 28 in the spleen and PLN. In agreement with our previous results, we observed a statistically significant reduction in Ki67 expression in CD73KO CD8+ T cells in PLN (Figure 1F). All these data suggest that CD73 promotes naïve CD8+ T cell homeostatic proliferation in lymphopenic mice.

As naïve T cells depend on IL-7/IL-7R signaling for survival and homeostatic proliferation, we studied CD127 (IL-7R alpha chain) expression in transferred CD73KO CD8+ T cells at day 10 and 28 following adoptive transfer into Rag1–/– recipient mice. As shown in Figure 1G, we observed a significant reduction in CD127 expression in CD73KO CD8+ T cells in the spleen and blood compared to WT cells at day 10. At day 28, we observed significant differences in CD127 expression between CD73KO and WT cells only in spleen. To study whether adenosine prevents the downregulation of CD127, we cultured naïve CD8+ T cells with NECA, an adenosine analog. Our results indicate that NECA prevented CD127 downregulation in WT cells following antigenic stimulation (Supplementary Figure 1). These findings suggest that CD73 and adenosine prevent CD127 downregulation on naïve CD8+ T cells and in consequence regulate their homeostatic proliferation.

### CD73 Does Not Affect Central Memory CD8+ T Cell Differentiation and Survival in Homeostatic Conditions

It has been shown that upon transfer into Rag1–/– mice, naïve CD8+ T cells proliferate and differentiate directly into memory cells (Cho et al., 2000). So next, we analyzed whether *in vivo* memory differentiation in lymphopenic mice is regulated by CD73. For this, after naïve CD8+ T cell transfer to Rag1–/– mice, we analyzed CD44 and CD62L expression in CD73KO and WT CD8+ T cells in the blood at different time points. As shown in Figure 1H, 7 days following the transfer, CD8+ T cells acquire a central memory (CD44+/CD62L+) and effector memory (CD44+/CD62L–) phenotype. By day 14, most cells present an effector memory phenotype, and this phenotype is maintained at later time points. Interestingly, the frequency of cells expressing a central memory phenotype was increased in CD73KO cells compared to WT CD8+ T cells at days 7, 14, and 21.

**Figure 2:**
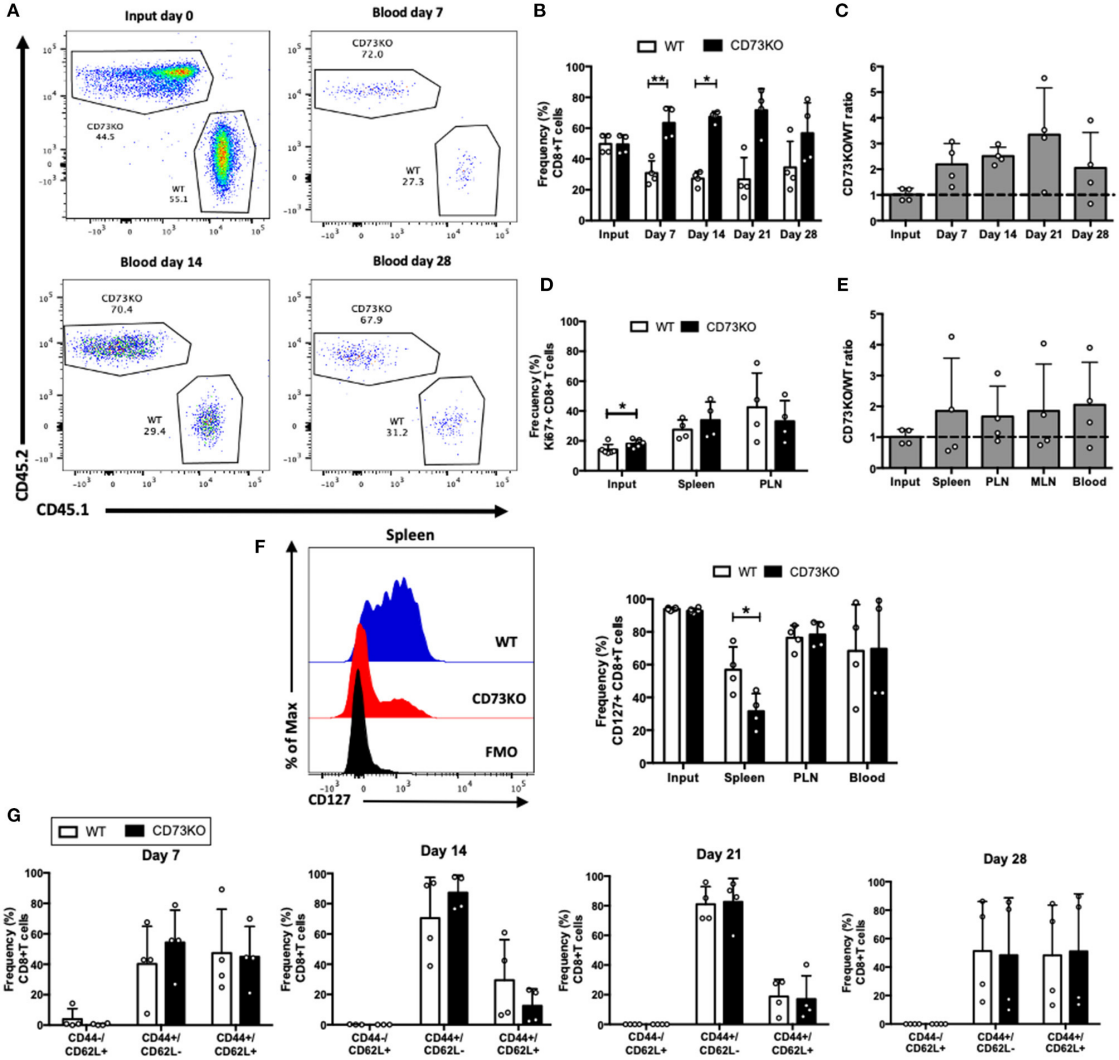
CD73 has no effect on central memory CD8+ T cell differentiation and survival in homeostatic conditions. Central memory CD8+ T cells (CD8+/CD25–/CD44+/CD62L+) obtained from WT (CD45.1+) and CD73KO (CD45.2+) mice were co-transferred in Rag1–/– mice. Rag1–/– mice were bled at different time points to analyze the frequency and phenotype of transferred cells. Mice were euthanized on day 28 to analyze the transferred cells in the spleen, PLN, and mesenteric lymph node (MLN). **(A)** FACS plots showing the input and frequency of transferred cells at days 7, 14, and 28 in blood. **(B)** Frequency of transferred cells at input and in the blood at days 7, 14, 21, and 28. **(C)** CD73KO/WT ratio at input and days 7, 14, 21, and 28 in the blood. **(D)** Frequency of Ki67+ cells at input, spleen and PLN at day 28. **(E)** CD73KO/WT ratio at input and day 28 in the spleen, PLN, MLN, and the blood. **(F)** Left, FACS histogram overlay depicting CD127 expression in transferred cells in the spleen at day 28. Right, frequency of CD127+ transferred cells at input and in the spleen, PLN and the blood at day 28. **(G)** Frequency of transferred cells expressing naïve (CD44–/CD62L+), effector memory (CD44+/CD62L–), and central memory (CD44+/CD62L+) phenotypes at days 7, 14, 21, and 28 in the blood. All data represent mean ± s.d. Data were analyzed by two-tailed unpaired Student's *t*-Test **(B)**, Mann–Whitney Test **(D,F,G)**, or one-way ANOVA with Bonferroni *post-hoc test* **(C,E)**. *p < 0.05; **p < 0.01.

To further investigate the role of CD73 on central memory cell survival, we co-transferred sorted CD73KO and WT central memory CD8+ T cells into Rag1–/– recipient mice and analyzed their frequency at different time points. As shown in Figures 2A,B, the frequency of CD73KO cells in the blood was higher than WT cells at early time points (day 7 and day 14), but this difference is abrogated at later time points (day 28). We observed no significant differences in CD73KO/WT ratio in the blood at all time points (Figure 2C). These data suggest that in contrast to naïve cells, CD73 only has an effect reducing the frequency of central memory CD8+ T cell at early time points in blood but does not impact the ratio of the transferred cells. On day 28, we observed no differences in Ki67 expression (Figure 2D), suggesting that any difference in T cell proliferation may be happening early following T cell transfer. The higher frequency of CD73KO CD8+ T cells observed on days 7 and 14 is not due to preferential migration of memory cells in lymph nodes since CD73KO/WT ratio was conserved among different organs (Figure 2E).

Interestingly, when we analyzed CD127 expression, we observed a reduction in the frequency of CD127+ T cells in CD73KO cells compared to WT cells in the spleen (Figure 2F), similar to what we observed for naïve CD8+ T cells. We did not find any differences on the frequencies of cells presenting an effector memory or central memory phenotype (Figure 2G). In summary, these data put forward the idea that CD73 prevents CD127 downregulation on central memory and naïve CD8+ T cells.

### CD73 Reduces CD8+ T Cell Maintenance Following Antigenic Stimulation

**Figure 3:**
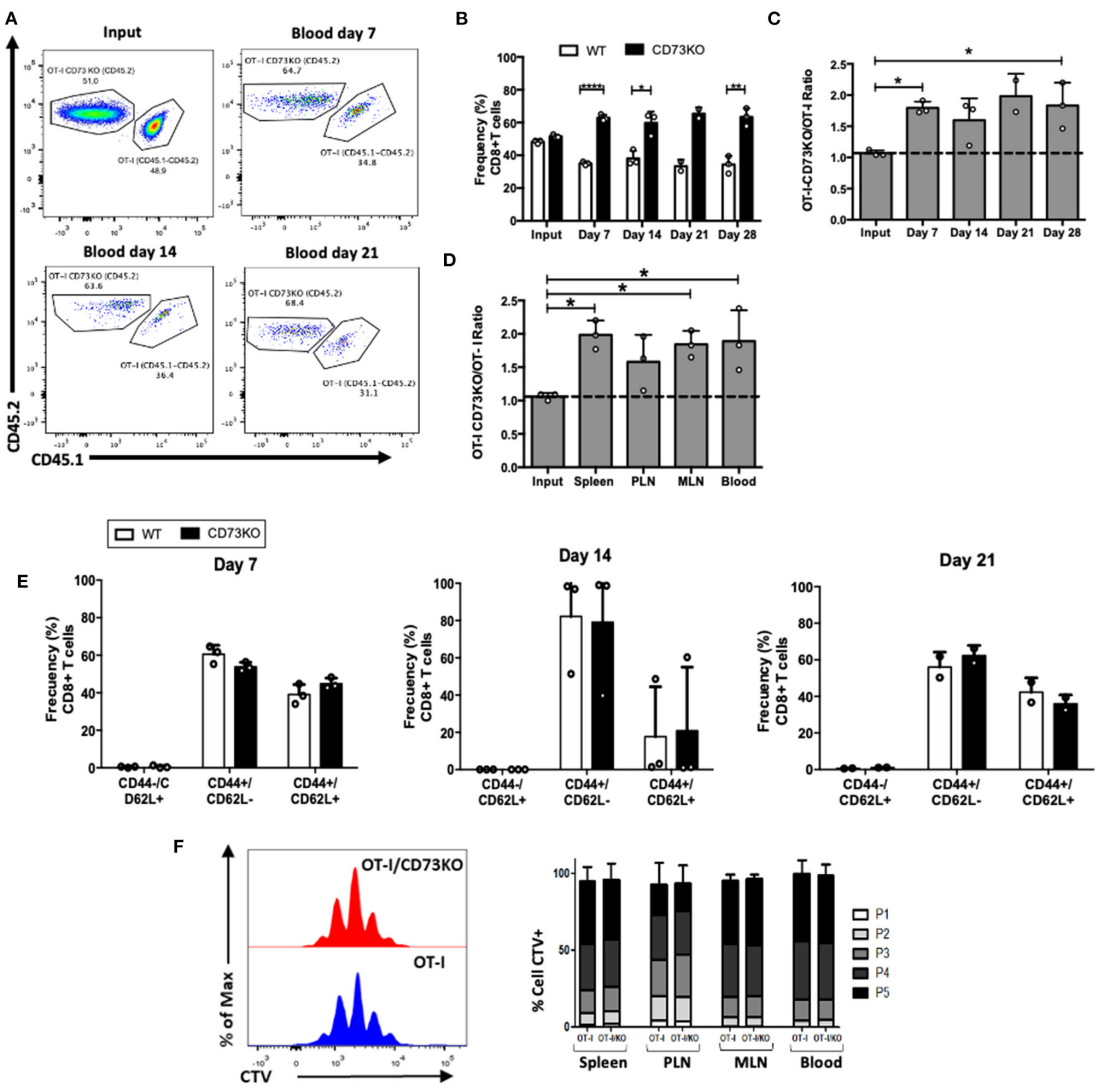
CD73 reduces the frequency of CD8+ T cells following *in vivo* antigenic stimulation. Naïve CD8+ T cells (CD8+/CD25–/CD44-/CD62L+) obtained from OT-I (CD45.1+/CD45.2+) and OT-I/CD73KO (CD45.2+) mice were co-transferred in CD45.1+ mice. Twenty-four hours later, recipient mice were immunized i.p. with OVA protein (500 μg) plus Poly I:C (50 μg). Mice were bled at different time points to analyze the frequency and phenotype of transferred cells. Mice were euthanized on day 28 to analyze the transferred cells in the spleen, PLN, and MLN. **(A)** FACS plots showing the input and frequency of transferred cells at days 7, 14, and 21 in blood. **(B)** Frequency of transferred cells in the blood at input and days 7, 14, 21, and 28. **(C)** CD73KO/WT ratio at input and days 7, 14, 21, and 28 in the blood. **(D)** CD73KO/WT ratio at input and day 28 in the spleen, PLN, MLN, and the blood. **(E)** Frequency of transferred cells expressing naïve (CD44-/CD62L+), effector memory (CD44+/CD62L-), and central memory (CD44+/CD62L+) phenotypes at days 7, 14, and 21 in blood. **(F)** Histogram overlay for CTV dilution and bar graph depicting the frequency of transferred cells in each proliferation round in the spleen, PLN, MLN, and blood. Naïve CD8+ T cells (CD8+/CD24–/CD44–/CD62L+) obtained from OT-I (CD45.1+/CD45.2+) and OT-I/CD73KO (CD45.2+) mice were labeled with cell trace violet (CTV) and co-transferred in CD45.1+ mice. Twenty-four hours later, recipient mice were immunized i.p. with OVA protein (500 μg) plus LPS (25 μg). Mice were euthanized on day 4 to analyze the proliferation of transferred cells as assessed by CTV dilution in the spleen, PLN, MLN, and the blood. All data represent mean ± s.d. Data were analyzed by two-tailed unpaired Student's *t*-Test **(B)**, Mann–Whitney Test **(E,F)**, or one-way ANOVA with Bonferroni *post-hoc test* **(C,D)**. *p < 0.05; **p < 0.01; ****p < 0.001.

Our data so far suggests that CD73 promotes naïve CD8+ T cell homeostatic proliferation, probably through a CD127 mediated mechanism. We asked whether this was also the case following antigenic stimulation of CD8+ T cells. For this, we co-transferred naïve antigen-specific (OT-I) CD73KO and WT naïve CD8+ T cells into CD45.1+ mice, and then mice were injected with their cognate antigen (OVA protein) plus Poly I:C to stimulate the *in vivo* activation of transferred cells. As shown in Figures 3A,B, the frequency of CD73KO CD8+ T cells was increased compared to WT cells in the blood at 7, 14, and 28 days. In agreement, CD73KO/WT cell ratio in the blood increased compared to the input at day 7 and 28 following transfer (Figure 3C). This increased ratio was also observed in the spleen and other lymph nodes again confirming that this difference is not due to an altered migration of CD73KO cells (Figure 3D). We observed no differences in the phenotype (CD44/CD62L expression) of transferred cells at different days following antigenic stimulation (Figure 3E).

To study whether this difference was due to increased proliferation rates on CD73KO cells, we co-transferred cell trace violet (CTV)-labeled antigen-specific CD73KO and WT cells and analyzed CTV dilution 4 days after OVA plus LPS injection in the blood and lymph nodes. As shown in Figure 3F, we found no differences in CD8+ T cell proliferation between CD73KO and WT cells. This data suggests that CD73 reduces the frequency of CD8+ T cells following *in vivo* antigenic stimulation and that this is probably not due to differences in the proliferative capacity between WT and CD73KO cells.

**Figure 4:**
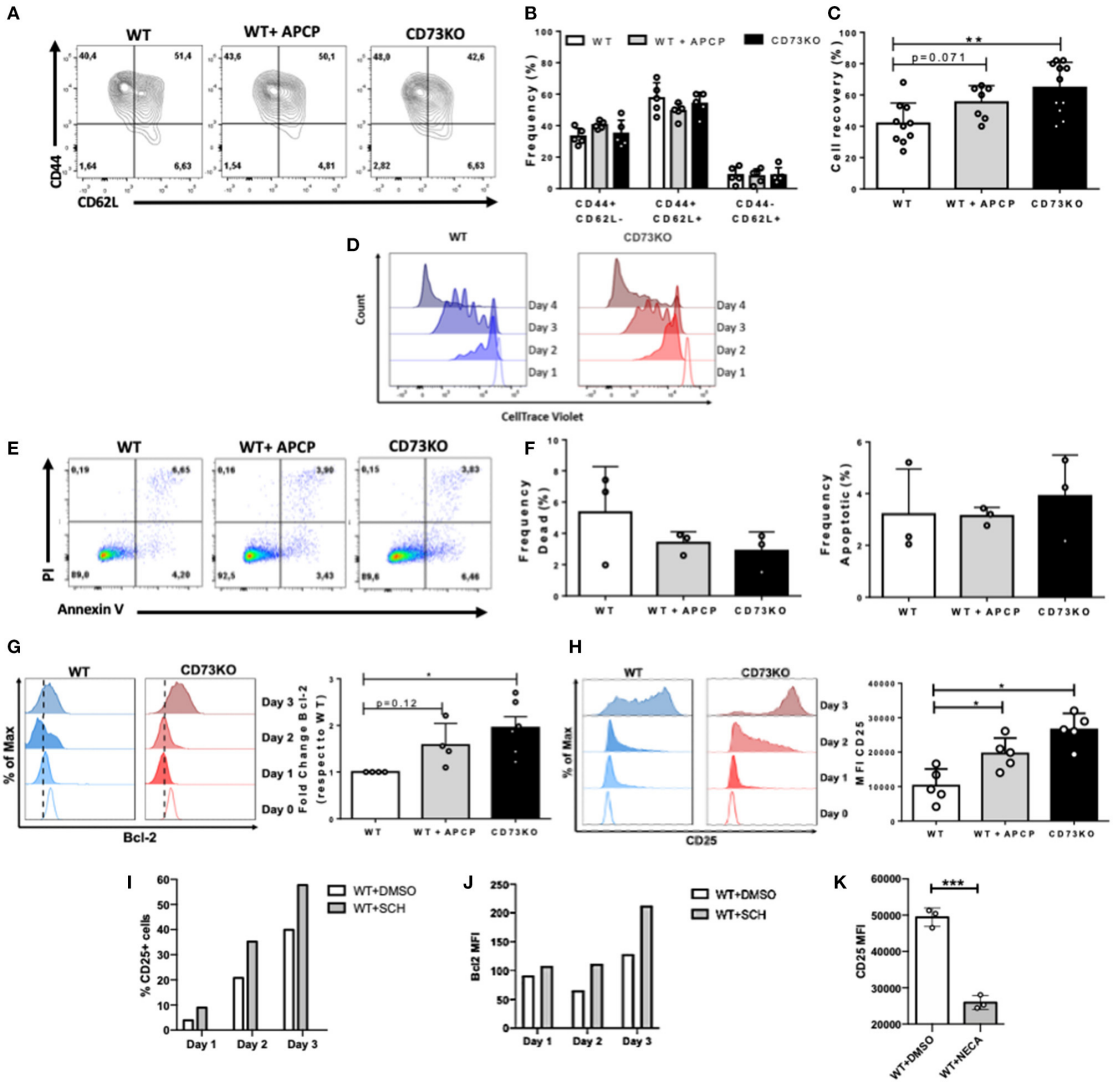
CD73 reduces CD25 expression in CD8+ T cells following *in vitro* antigenic stimulation. Sorted naïve CD8+ T cells from WT and CD73KO mice were labeled with CTV and activated *in vitro* with antibodies against CD3/CD28 in the presence of IL-2 (10 ng/ml). Where indicated, APCP (50 μM), SCH58261 (5 μM), or NECA (1 μM) were added to the cultures. At different time points, cells were harvested and analyzed by FACS. **(A)** FACS plot depicting CD62L and CD44 expression in WT cells, APCP-treated WT cells, and CD73KO cells at day 4 following activation. **(B)** Frequency of cells expressing CD62L and CD44 for WT cells, APCP-treated WT cells, and CD73KO cells at day 4 following activation. **(C)** Percentage of cell recovery after 4 days of activation for WT cells, APCP-treated WT cells, and CD73KO cells. Percentage of cell recovery: (absolute number of cells obtained after culture/ absolute number of cells cultured at the start of the assay) × 100. **(D)** Histogram overlay depicting proliferation (as cell trace violet dilution) for WT and CD73KO cells at different time points following activation (representative of three independent experiments). **(E)** FACS plot showing PI/Annexin V staining following 4 days of activation for WT cells, APCP-treated WT cells, and CD73KO cells. **(F)** Frequency of dead and apoptotic cells recovered after 4 days of activation for WT cells, APCP-treated WT cells, and CD73KO cells. **(G)** Left, histogram overlay showing Bcl-2 expression at different time points for WT and CD73KO cells. Right, Bcl-2 expression fold-change (relative to WT cells) in CD73KO cells and APCP-treated WT cells after 3 days of activation. **(H)** Left, histogram overlay depicting CD25 expression at different time points for WT and CD73KO cells. Right, bar graph showing CD25 mean fluorescence intensity (MFI) following 3 days of activation for WT, CD73KO and APCP-treated cells. (I) Percentage of CD25+ cells for WT cells at different time points following activation in the presence of SCH58261 or its vehicle control (DMSO). **(J)** Bcl2 MFI for WT cells at different time points following activation in the presence of SCH58261 or its vehicle control (DMSO). **(K)** CD25 MFI for WT cells at day 4 following activation in the presence of NECA or its vehicle control (DMSO). All data represent mean s.d. Data were analyzed by the Kruskal-Wallis test with Dunn's *post-hoc* test **(B,C,F–H)** and by two-tailed unpaired Student's *t*-Test **(K)** *p < 0.05; **p < 0.01, ***p < 0.005.

To further investigate the mechanisms that explain this increase in the frequency of CD73KO cells following *in vivo* antigenic stimulation, we performed *in vitro* activation assays. For this, naïve CD8+ T cells from WT and CD73KO mice were sorted, labeled with CTV, and stimulated with soluble anti-CD3/CD28 antibodies in the presence of IL-2 for 3-4 days. To block CD73 enzymatic activity, we treated WT cells with the CD73 inhibitor APCP [Adenosine 5'-(a,b-methylene) diphosphate] (50 μM). As shown in Figure 4A, after antigenic stimulation, a high percentage of cells upregulated CD44 expression, but we found no differences in the frequencies of CD44+/CD62L+ or CD44+/CD62L- cells between CD73KO, WT, or APCP-treated WT cells (Figures 4A,B). However, following *in vitro* activation, we obtained a higher percentage of cell recovery in CD73KO cultures than WT cells (Figure 4C). This difference was not due to a higher proliferative response of CD73KO cells since we observed a slight delay in the proliferative response of CD73KO CD8+ T cells (Figure 4D). Although we found no differences in the frequency of apoptotic or dead cells in these cultures (Figures 4E,F), we found a 1.9-fold increase in Bcl-2 expression in the CD73KO and 1.5-fold increase for APCP-treated cells compared to WT cells (Figure 4G). Interestingly, we also observed a significant increase in CD25 (IL-2R alpha chain) expression on CD73KO and APCP-treated WT cells compared to WT cells (Figure 4H). Accordingly, CD25 and Bcl2 expression in WT CD8+ T cells was upregulated in the presence of a selective A2AR antagonist SCH58261 following antigenic stimulation (Figures 4I,J), whereas CD25 expression was downregulated by NECA (Figure 4K), demonstrating that adenosine signaling is negatively regulating Bcl2 and CD25 expression in CD8+ T cells. These data support the idea that CD73 and adenosine reduces CD8+ T cell survival following antigenic stimulation by restraining Bcl-2 and CD25 expression.

## Discussion

CD73 is the main enzyme involved in the generation of extracellular adenosine. This nucleoside has significant suppressive effects on the immune system, including the inhibition of the proliferation and function of antigen-specific cytotoxic T cells (Ohta et al., 2006; Vigano et al., 2019; Feng et al., 2020). Consequently, both adenosine and the ectonucleotidases responsible for its production are currently considered targets for antitumor therapies. In this line, new therapies using blocking antibodies and small molecules against CD73 have been suggested (Antonioli et al., 2017), highlighting the relevance of studying CD73 function on CD8+ T cells. Here we analyzed the role of CD73 on CD8+ T cell survival and expansion under antigenic and homeostatic conditions. We show that CD73 has a dual effect on naïve CD8+ T cells. On one side, favoring interleukin-7 receptor α chain expression and homeostatic survival of CD8+ T cells; while reducing interleukin-2 receptor α chain, Bcl-2 expression, and the survival of CD8+ T cells under antigenic stimulation. In contrast to naïve CD8+ T cells, CD73 did not impact central memory CD8+ T cells' accumulation under homeostatic conditions. All these data suggest that CD73 blockade in the context of cellular therapies against cancer might not only reduce adenosine production by tumor cells but also impact the survival of naïve CD8+ T cells.

When evaluating the role of CD73 in the homeostatic proliferation of naïve CD8+ T cells in lymphopenic Rag1–/– animals, we observed that the frequency of CD73KO cells decreased over time compared to WT cells, suggesting that CD73 may promote the survival of naïve CD8+ T cells in this context. This observation agrees with our previous evidence demonstrating a crosstalk between adenosine and IL-7 signaling, where adenosine has been shown to induce CD127 expression through A2A receptor signaling (Cekic et al., 2013). Moreover, our recent findings indicate that IL-7 signaling has been shown to support CD8+ T cell maintenance despite the presence of extracellular adenosine (Koyas et al., 2021). Thus, it is possible to speculate that the decreased frequency of CD73KO cells under homeostatic conditions may be explained by defective A2AR signaling that translates into a reduced CD127 expression and weak IL-7 signaling for the maintenance of these cells.

Although we report an accumulation of WT cells in our homeostatic proliferation assays, we observed no difference in Ki67 staining at day 28 between CD73KO and WT cells. This may be because the cells appear to be in a contraction phase at the time point analyzed. However, we could not detect substantial Ki67 expression at early time points (day 10 after adoptive transfer). Further experiments are needed to detect the exact timing of T cell proliferation under these homeostatic conditions.

In contrast to naïve cells, we observed that the frequency of central memory cells was increased in the absence of CD73 only at early time points but CD73KO/WT ratio was not affected. In the case of central memory cells, we also observed a reduction in CD127 expression in CD73KO cells compared to WT cells. Although IL-7 and IL-15 have been described as crucial for T cell homeostasis, IL-15 is considered the critical cytokine supporting the homeostatic proliferation of memory cells (Lodolce et al., 1998; Becker et al., 2002; Raeber et al., 2018). Thus, in the case of central memory CD8+ T cells, CD127 might be dispensable for their homeostatic proliferation, and other signaling pathways triggered by IL-15 might become relevant (Hand et al., 2010; Kim and Suresh, 2013).

In contrast to our observation in the homeostatic conditions, adoptive transfer experiments of naïve CD8+ T cells from OT-I and OT-I/CD73KO mice in WT animals demonstrated that, in the presence of strong antigenic stimulation, CD73 expression is deleterious to lymphocytes as it reduces their survival. When analyzing whether this effect is due to changes in the proliferation rate, we demonstrated that OT-I and OT-I/CD73KO T cells do not show differences in the number of proliferation cycles in early stages *in vivo*. These results strongly suggest that CD73 may be reducing the survival of the transferred CD8+ T cells following antigenic stimulation. This agrees with our data from *in vitro* experiments where we observed an increase in the expression of the anti-apoptotic protein Bcl-2 and the IL-2R alpha chain (CD25) in the CD73KO cells. It has been reported that Bcl-2 is critical in the survival of effector cells (Kurtulus et al., 2011) and that its expression is upregulated by IL-2 through the PI3K/Akt signaling pathway (Kelly et al., 2002). Therefore, one explanation for the enhanced survival of CD73KO CD8+ T cells that we report may be related to a stronger IL-2 signaling, which leads to a higher Bcl-2 expression and decreased apoptosis.

As previously stated, following antigenic stimulation of naïve CD8+ T cells, we observed a more potent CD25 upregulation in CD73KO T cells, and APCP-treated WT cells compared to WT cells. It is well-recognized that CD25 and IL-2 signaling promotes terminal effector differentiation of CD8+ T cells (Kalia et al., 2010). Therefore, under a strong antigenic stimulus, CD73 and adenosine could be inhibiting the differentiation of CD8+ T lymphocytes to an effector phenotype. In this line, our results suggest that CD73-deficient cells present a higher cytotoxic potential, evidenced by an increase in IFN-γ, TNF-α, and granzyme B production. Moreover, CD73-deficient cells presented an increased glucose uptake and higher mitochondrial respiration, indicating that this ectonucleotidase restricts the mitochondrial capacity in CD8+ T cells. Finally, when adoptively transferred into tumor-bearing mice, CD73 deficient cells were more effective against the tumor and expressed lower levels of exhaustion markers (Briceño et al., 2021). All this data suggests that CD73 may act as a checkpoint inhibitor under antigenic stimulation, limiting effector differentiation and delaying cytotoxic T cells' metabolic reprogramming.

**Figure 5:**
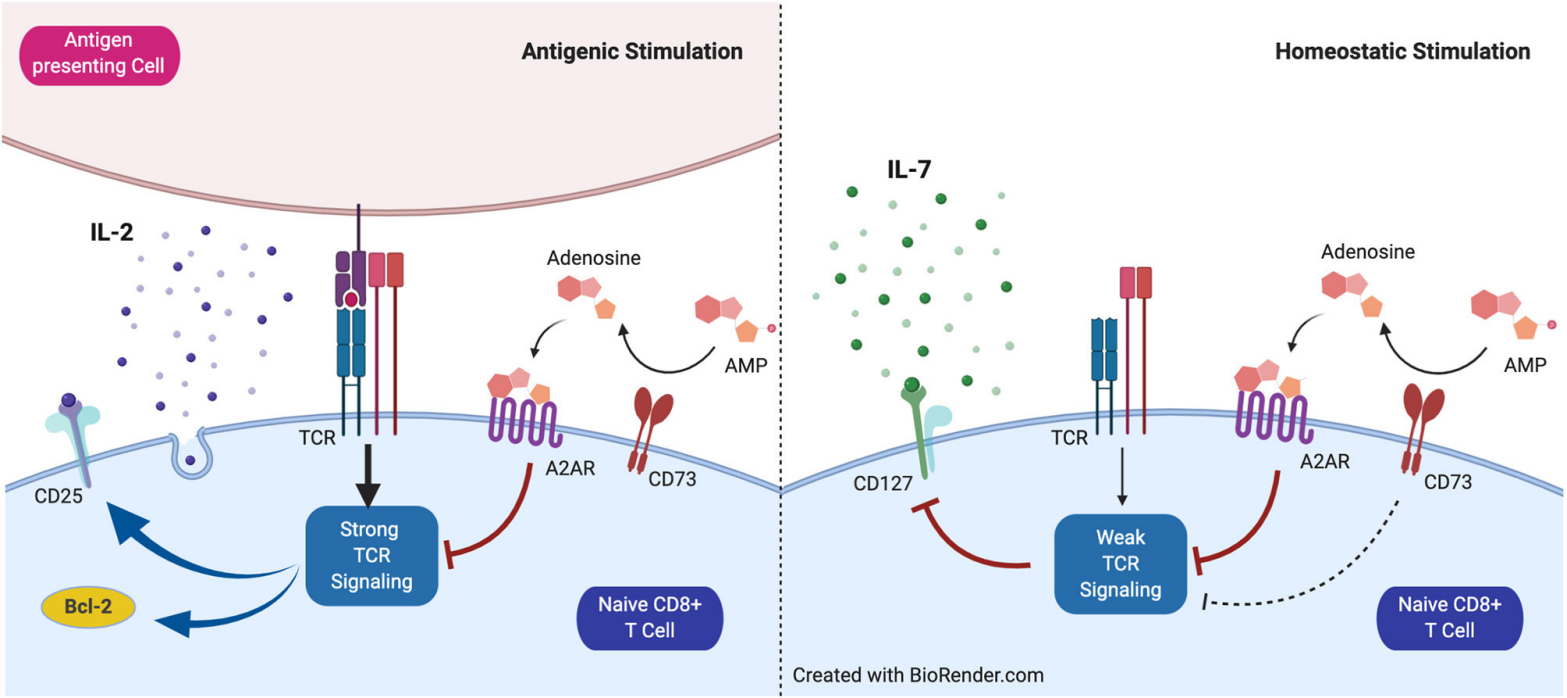
Proposed model for the role of CD73 in homeostatic and antigenic stimulation of naïve CD8+ T cells. CD73 reduces signaling mediated by the T cell receptor (TCR) and restrains CD25 and Bcl-2 expression under antigenic stimulation. Under homeostatic conditions, CD73 prevents CD127 downregulation mediated by weak TCR stimulation.

The results presented here allow us to propose a model in which CD73 has a dual role in the survival of CD8+ T cells (Figure 5). When faced with a condition of strong TCR activation, the expression of CD73 confers a disadvantage to CD8+ T cells. This is associated with a decrease in the survival and a lower effector capacity of CD8+ T cells, which is manifested by the reduction of Bcl-2 and CD25 expression. On the other hand, under conditions of weak TCR activation, such as during homeostatic proliferation in the presence of IL-15 and IL7, the expression of CD73 confers an advantage for the survival of CD8+ T cells, through the prevention of CD127 downregulation. The contribution of the catalytic function of this ectonucleotidase and the function of CD73 as a molecule associated with signal transduction in the differentiation of T lymphocytes remains to be elucidated.

Currently, the role of adenosine in the antitumor response, through its receptors and the action of CD73, is being actively studied. Our study acquires relevance in light of the possible use of CD73 inhibitors in adoptive cell therapies since they are usually accompanied by a prior lymphoconditioning, either by chemotherapy or radiation. This prior lymphoconditioning provides advantages for the performance of adoptive cell therapies since it eliminates regulatory T cells and other cells that suppress the immune response in the tumor and increases the levels of IL-7 and IL-15 available for the transferred cells, recreating homeostatic conditions (Gattinoni et al., 2006; Restifo et al., 2012; Hinrichs and Rosenberg, 2014). We conclude that special attention should be given to CD73 blockade since its inhibition could be beneficial by increasing CD8+ T cells' effector capacity but detrimental during the initial homeostatic phase following the lymphoconditioning process.

## Data Availability Statement

The original contributions presented in the study are included in the article/Supplementary Material, further inquiries can be directed to the corresponding author/s.

## Ethics Statement

The animal study was reviewed and approved by CICUA-Universidad de Chile.

## Author Contributions

MVR and ST performed experiments and analyzed the data. BP-T, PB, ER-Y, JS-A, PH, and BM performed experiments, analyzed the data, and wrote the manuscript. AL, CC, DS, MB, and MR designed the study and wrote the manuscript. All authors critically read the manuscript.

## Conflict of Interest

The authors declare that the research was conducted in the absence of any commercial or financial relationships that could be construed as a potential conflict of interest.
