# Supplementary figures and images for: Ecto-5′-Nucleotidase (CD73) Regulates the Survival of CD8+ T Cells
Source: Front Cell Dev Biol. 2021 Apr 13;9:647058. doi: 10.3389/fcell.2021.647058 (PMC8076893; doi:10.3389/fcell.2021.647058)

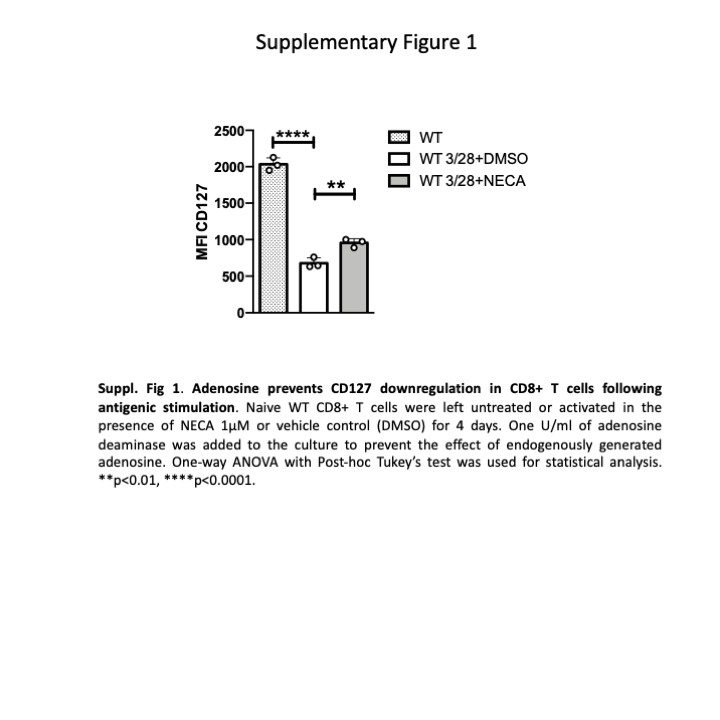

Supplement: Supplementary file 1 [file Image_1.TIFF]
